# Structural and biophysical characterization of the multidomain xylanase Xyl

**DOI:** 10.1371/journal.pone.0269188

**Published:** 2022-06-03

**Authors:** Valentine Anye, Robert F. Kruger, Wolf-Dieter Schubert

**Affiliations:** Department of Biochemistry, Genetics and Microbiology, Faculty of Natural and Agricultural Sciences, University of Pretoria, Pretoria, South Africa; Weizmann Institute of Science, ISRAEL

## Abstract

The depletion of fossil fuels, associated pollution, and resulting health hazards are of concern worldwide. Woody biomass constitutes an alternative source of cleaner and renewable energy. The efficient use of woody biomass depends on xylan depolymerisation as the endo-β-1,4-xylopyranosyl homopolymer is the main component of hemicellulose, the second most abundant component of wood. Xylan depolymerisation is achieved by hemicellulolytic xylanases of glycoside hydrolase (GH) families 5, 8, 10, 11, 30 and 43 of the CAZY database. We analysed a multidomain xylanase (Xyl) from the hindgut metagenome of the snouted harvester termite *Trinervitermes trinervoides* that releases xylobiose and xylotriose from beech and birch xylan and wheat arabinoxylan. The four domains of Xyl include an N-terminal GH11 xylanase domain, two family 36-like carbohydrate-binding domains CBM36-1 and 2, and a C-terminal CE4 esterase domain. Previous analyses indicated that CBM36-1 deletion slightly increased GH11 catalysis at low pH whereas removal of both CBMs decreased xylanase activity at 60°C from 90 to 56%. Possible cooperativity between the domains suggested by these observations was explored. A crystal structure of the two-domain construct, GH11-CBM36-1, confirmed the structure of the GH11 domain whereas the CBM36-1 domain lacked electron density, possibly indicating a random orientation of the CBM36-1 domain around the GH11 domain. Isothermal titration calorimetry (ITC) experiments similarly did not indicate specific interactions between the individual domains of Xyl supporting a “beads-on-a-string” model for Xyl domains.

## Introduction

Mineralised organic compounds of biological origin remain the primary source of energy for human economies. The steady depletion and adverse environmental effects of fossil fuel utilization have spurred the search for renewable energy forms. Biofuels from abundant and renewable materials could provide an alternative to the currently rapidly depleting fossil fuels [1]. First-generation, food crop-based biofuels from grains, oilseeds, sugar cane or sugar beet raise socio-economic and environmental concerns due to food security, scarcity of water and arable land, as well as their impact on ground water and soil quality [2, 3]. Second generation biofuels are derived from non-food biomass such as agricultural and forest residues rich in lignocellulose [4, 5]. Their depolymerisation yields useful sugar components yet remains underutilised due to limited success in adapting to industrial processes [6, 7]. This bottleneck may potentially be overcome by combining multiple lignocellulose hydrolysing enzymes or a single enzyme with multiple catalytic domains capable of transforming complex polysaccharides into industrially useful fermentable sugars [8–10]. Xylanases depolymerise hemicellulose, the second most abundant component of lignocellulosic biomass, into simple sugars useful in biofuel production. Understanding the structure-function relationship of xylanases and their accessory enzymes may contribute to efficient lignocellulose depolymerisation.

**Fig 1:**
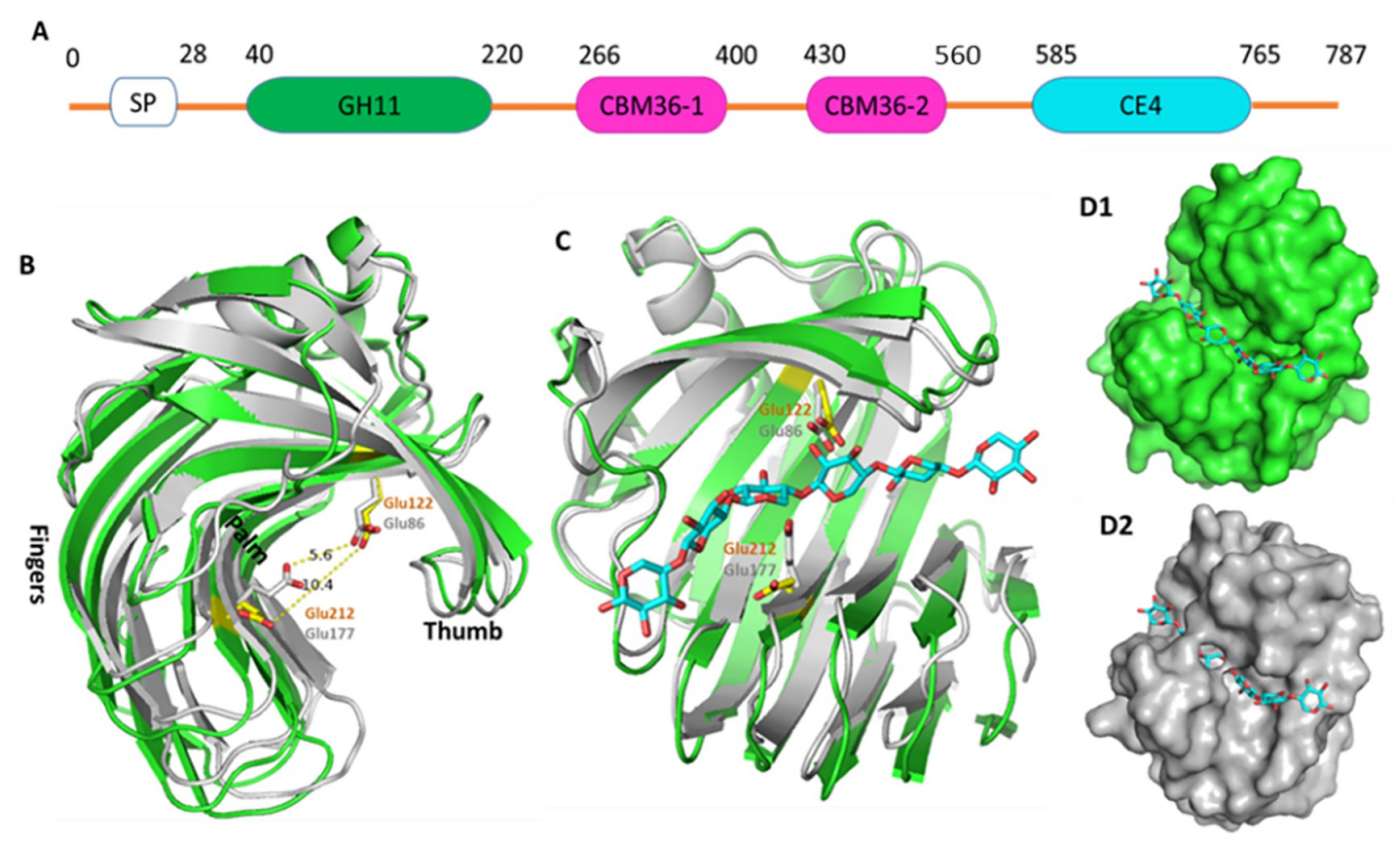
Domain structure of Xyl and crystal structure of the Xyl-GH11. **A)** An N-terminal secretion signal peptide (SP) is followed by a GH11 catalytic domain (green), two carbohydrate binding modules CBM36-1 and CBM36-2 (magenta), and an esterase CE4 catalytic domain (cyan). **B)** Crystal structures of Xyl-GH11 (green) superimposed on the crystal structure of TrXyn11 (gray) showing the “fingers” and “palm” (outer and inner β-sheets), and the “thumb” loop. The catalytic residues are shown as sticks. Glu177 of TrXyn11 adopts an “up” conformation (gray) whereas the corresponding residue Glu212 in Xyl-GH11 is in the “down” conformation (yellow). **C)** Xylohexose modelled into the active site of Xyl-GH11 and TrXyn11. Surface view shows the modelled substrate is enclosed by the enzyme in TrXyn11 with the up conformation **(D2**) but open in Xyl-GH11 (**D1**).

Termites degrade and utilise up to 95% of the cellulose and 80% of the hemicellulose components of lignocellulose. Termite microbiota thus provide an attractive source of enzymes for lignocellulose biofuel production [11]. The snouted harvester termite *Trinervitermes trinervoides* uses enzymes from different gastrointestinal symbionts to digest polysaccharides [12]. We investigated the xylanase Xyl from the hindgut metagenome of the snouted harvester termite. Xyl consists of a GH11 catalytic domain (Xyl-GH11), two family 36-like carbohydrate binding modules (Xyl-CBM36) and a C-terminal family 4 carbohydrate esterase (Xyl-CE4) domain (Fig 1A). An N-terminal signal peptide (SP) removed during secretion was excluded from this study.

CBM36 domains are not known for direct interactions with other protein domains [13]. An increase or decrease in GH11 catalysis observed following the deletion of CBM36-1 or the removal of both CBM36-1 and -2 in our previous study [14], raised the question of whether CBM36 domains did interact with other domains after all. Could CBM36 domains enhance catalytic domain—substrate interactions beyond a linker-based co-localization? This study expanded the investigation of Xyl and its domains using X-ray crystallography and isothermal titration calorimetry (ITC).

## Results and discussion

### General statement of research

To produce bioethanol from lignocellulose, the latter needs to be depolymerised ideally by a set of suitable enzymes [15, 16]. While protein domains mostly have distinct functions [17], the combination of domains in multidomain proteins and the interaction of these domains with each other, with ligands or with other biological structures determine their overarching function. We examined individual domains of the multidomain protein Xyl (NCBI GenBank accession number **AMO13186**) identical to the endo-1,4-β-xylanase from *Butyrivibrio* sp. XPD200 from the hindgut metagenome of *Trinervitermes trinervoides* [12] focusing on the structure, function, and possible interaction of the domains. The full-length enzyme was successfully produced and purified but proved prone to degradation. Instead, individual domains and domain combinations were investigated structurally and for possible interdomain interactions. This analysis involved two catalytic domains, N-terminal GH11 and C-terminal CE4, as well as two central, carbohydrate binding domains CBM36-1 and 2. SDS PAGE analysis of full-length Xyl mostly revealed a band equivalent in size to the GH11-2CBM36 construct as well as smaller fragments due to further cleavage (S1 Fig 1 in S1 File). Individual domains and shorter domain combinations were significantly more soluble and stable than the full-length protein.

### Structural characterization of the GH11 domain of Xyl

The previous partial characterisation of the Xyl-GH11 domain [14] was expanded by structural analysis. Crystals were obtained using 10 mg/mL Xyl-GH11 in 50 mM Na acetate pH 4.8, 500 mM (NH_4_)_2_SO_4_ at 18°C. Diffraction data were collected on beamline ID29 (ESRF, Grenoble, France) and processed using XDS [18] (Table 1). The structure was solved by molecular replacement using the crystal structure of a xylanase from *Bacillus* sp. 41M-1 (PDB ID: **2DCJ,** unpublished) as a model. Xyl-GH11 coordinates were deposited in the protein data bank (PDB ID **7ZSZ)**.

**Table 1:** Data collection and refinement statistics.

Data collection statistics	GH11	GH11-CBM36-1	CBM36-1	CE4
Wavelength	0.976 Å	0.976 Å	1.54 Å	1.54 Å
Space group	P1	P6_3_	P2_1_2_1_2_1_	P2_1_2_1_2_1_
Unit cell: a b c (Å) α β γ (°)	38.5 43.4 69.9 100.9 96.3 116.0	108.2 108.2 54.2 90 90 120	28.4 37.3 108 90 90 90	40.1 59.7 86.1 90 90 90
Resolution range* (Å)	38.5 43.4 69.9 100.9 96.3 116.0	93.69–1.47 (3.90–1.47)	25.16–1.99 (2.06–1.99)	23.07–1.93 (2.12–2.05)
Total reflections*	38.5 43.4 69.9 100.9 96.3 116.0	353537 (11941)	51380 (2092)	109092 (2558)
Unique reflections*	68762 (2652)	65364 (3225)	8260 (521)	13512 (1307)
Multiplicity*	1.6 (1.6)	5.4 (3.7)	6.3 (4.0)	7.1 (6.6)
R_merge_	0.049 (0.278)	0.051 (0.473)	0.055 (0.102)	0.029 (0.086)
Completeness* (%)	87.5 (67.0)	99.9 (99.0)	98.3 (87.8)	99.0 (96.9)
I/Isig	13.6 (2.1)	15.8 (2.2)	22.0 (9.4)	41.1 (17.9)
**Refinement statistics**				
R-work* R-free*	0.181 (0.216) 0.217 (0.268)	0.171 (0.199) 0.187 (0.252)	0.175 (0.219) 0.249 (0.294)	0.173 (0.253) 0.243 (0.372)
Ramachandran: favoured, additional, outliers (%)	97.5, 2.5, 0.0	97.0, 3.0, 0.0	95.9, 3.3, 0.8	97.5, 0.5, 2.0
Rotamer outliers (%)	1.16	0.58	0.00	1.66
Clash score	3.08	1.94	1.11	2.83
RMS bonds (Å), angles (°)	0.006, .886	0.006, 0.934	0.006, 0.785	0.006, 0.708
PDB code	**7ZSZ**	**7AYP**	**7AY3**	**7AX7**

* Values in parentheses are for highest-resolution shell.

The Xyl-GH11 domain crystallised in space group P1 diffracting X-rays to a resolution of 1.39 Å but yielding a low completeness of only 87.5 or 67.0% for the entire data set or the shell of highest resolution. This is a result of the low symmetry space group and a non-ideal crystal orientation, yet still reveals most critical structural details. The Xyl-GH11 domain was also resolved with 99.9% completeness (Table 1) from the GH11-CBM36-1 construct described below.

Two symmetrically independent Xyl-GH11 domains occupy the asymmetric unit. Each Xyl-GH11 adopts a β-jelly-roll fold composed of 14 β-strands, one α-helix and mostly short loops of up to five residues (Fig 1B). The β-strands form two curved, anti-parallel β-sheets stacked in a β-sandwich. The outer sheet is planar, but the inner one is bent creating a long, deep cleft. A single α-helix runs adjacent to the convex surface of the outer β-sheet. The domain resembles a partially closed right hand with the β-sheets forming palm and fingers while an extended loop between β-strands 8 and 9 forms the thumb (Fig 1B).

The substrate-binding cleft of Xyl-GH11 lies within the inner β-sheet cleft. Comparing Xyl-GH11 to related enzymes including the *Trichoderma reesei* enzyme Xyn11 (TrXyn11), identify Glu122 and Glu212 as catalytic nucleophile and general acid, respectively [19]. The latter adopts “up” and “down” conformations as part of its catalytic cycle. Here, the down position of Glu 212 of Xyl-GH11 is linked to an open conformation with 10.4 Å between two carboxylates (Fig 1D1) compared to 5.5 Å in catalytically active conformations (Fig 1B and 1D2).

### Carbohydrate binding modules of Xyl

**Fig 2:**
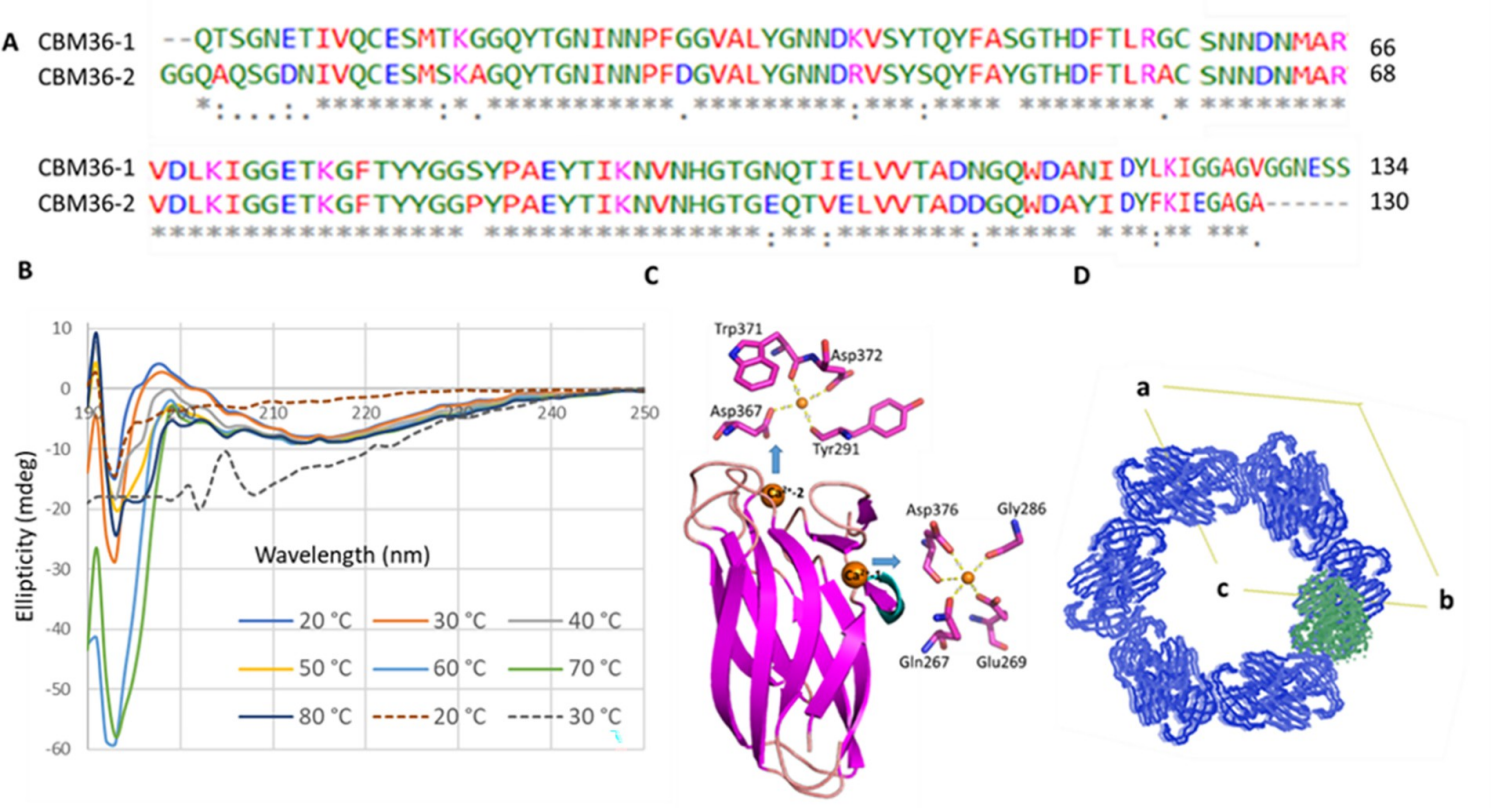
Characterization of Xyl-CBM36-1. **A)** Alignment of CBM36-1 and CBM36-2 with 84% sequence identity. Asterisks, colons, dots, and spaces mark conserved and progressively more divergent substitutions. Numbers are relative to the CBM36 domains. **B)** CD spectra of CBM36-1 from 20 to 80°C at 10°C intervals (continuous lines). The CBM36-1 structure is thermophilic remaining largely intact up to 80°C. CBM36-2 spectra (20 and 30°C) are shown as broken lines. The partially folded CBM36-2 protein at 20°C loses its fold at 30°C. 4 μM of each sample in 25 mM Tris pH 8.0, 25 mM NaCl was used for CD spectroscopy. **C)** Crystal structure of CBM36-1 domain of Xyl at 2.0 Å resolution showing two Ca^*2+*^ binding sites (orange spheres) and a lone α-helix (cyan). **D)** Packing analysis of GH11-CBM36-1 crystal structure: Only electron density for the GH11 molecule (green) was visible. With its symmetry related counterparts (blue), the GH11 molecule creates a cylindrical tunnel.

Xyl bears two family 36 carbohydrate binding modules (CBM) sharing 84% sequence identity (Fig 2A). CBM36-1 and -2 mainly differ in three residues: residue 50 (Tyr in -1 and Ser in -2), 86 (Pro/Ser) and 119 (Tyr/Asn) using relative residue numbering. Additional conservative substitutions are indicated in Fig 2A. Linkers N- and C-terminal to the CBM36 domains also differ slightly. Thus, the N-terminal linker of CBM36-2 includes two Gly residues not found in CBM36-1 and CBM36-1 has a C-terminal G-G-N-E-S-S sequence missing in CBM36-2. Gene fragments encoding the two domains were individually cloned, and proteins produced and purified. CD spectroscopy revealed CBM36-1 to be folded at 20°C and to retain its fold up to 80°C. CBM36-2, however, was only partly folded at 20°C and completely unfolded at 30°C (Fig 2B). CBM36-1 was crystallised in 0.1 M Hepes pH 7.5 and 25% PEG 3000. Attempts to crystallise CBM36-2 were unsuccessful. Why limited differences in the sequences of CBM36-1 and -2 would prevent the latter from folding remains unclear given that high quantities of soluble protein were obtained (S1 Fig 2 in S1 File).

The crystal structure of CBM36-1 (Fig 2C, PDB ID: **7AY3**) was solved at 2.0 Å resolution by molecular replacement using a family 36 carbohydrate-binding module (PDB ID: **1UX7**, [13]. CBM36-1 consists of 10 β-strands and a short α-helix between the first two β-strands. Eight β-strands form two 4-stranded, antiparallel β-sheets creating a jellyroll structure. Electron density for two metal ions were identified in CBM36-1 and modelled as Ca^2+^. Metal ion site 1 or Ca^2+^-1 is adjacent to the sole α-helix, while Ca^2+^-2 is in a depression at one end of the jellyroll (Fig 2C). Ca^2+^-1 is coordinated by main chain carbonyl oxygen atoms of Gly286 and Asp376, and the side chains of Gln267, Glu269 and Asp376, whereas Ca^2+^-2 is coordinated by carbonyl groups of Tyr291 and Trp371, and side chains of Asp367 and Asp 372 (Fig 2C).

CBM36 domains have been linked to protein stability and substrate binding [14, 20]. A structural alignment of CBM36-1 and the CBM36 domain of *Paenibacillus polymyxa* xylanase 43A (CBM36_Xyn43A; PDB ID **1UX7**) identifies the substrate binding site of CBM36-1 within a pocket around Ca^2+^-2. The pocket is lined with aromatic residues including Tyr277, Tyr293 and Trp371 for substrate binding. The flexible loop Ala366-Asp367-Asn368-Gly369 also contributes to ligand binding [13].

### GH11-CBM36-1 crystal structure

Tethering of catalytic and CBM domains provides distinct advantages in polysaccharide degradation: increase effective enzyme concentrations on the polysaccharide surface; target of catalytic module to the substrate; and disruption of the polysaccharide structure [21]. A two-domain construct, GH11-CBM36-1, previously cloned and biochemically characterised [14] was structurally analysed in this study. Single crystals were obtained after three months using 10 mg/ml protein in 50 mM Na acetate pH 4.8, 0.5 M (NH_4_)_2_SO_4_ at 18°C. Diffraction data were collected on beamline ID29 at the European Synchrotron Radiation Facility (ESRF), Grenoble, France and automatically processed. The structure was solved using structures of individual domains (see above) as models. The crystal structure of the GH11-CBM36-1 construct revealed electron density only for the GH11 domain and was refined to an R-free of 0.187 with a 99.9% completeness. This structure filled in the potentially missing information from the Xyl-GH11 domain above, which had a low completeness of 87.5%. Interestingly, the GH11 domains pack to create a hexagonal cylinder around the C-axis providing sufficient space for the CBM36-1 domains (Fig 2D), however, the CBM36-1 could not be located. While the lack of electron density for the CBM36-1 domain could imply that the linker between the domains was degraded during crystallization, this is unlikely as GH11 would then presumably have crystallized in the same triclinic packing observed for the Xyl-GH11 domain above. The hexagonal packing of GH11-CBM36-1 has a solvent content of 69% and a Matthews coefficient of 4 (if GH11 only is considered), while the triclinic packing has a solvent content of 38% and a Matthews coefficient of 1.97. Thus, CBM36-1 potentially adopts a random orientation relative to the GH11 domain within the central void of the GH11 cylindrical tunnel. Comparing the molecular packing of GH11-CBM36-1 to that of the only crystal structure combining a GH11 with a visible CBM36 domain in the PDB, Xynj (PDB ID: **2DCJ),** reveals that linker length could partly account for the random orientation of the CBM36-1 domain in GH11-CBM36-1. The 33-residue linker between GH11 and CBM36-1 is three times longer than the linker between the two domains in Xynj. Thus, although XynJ also crystallised in a cylindrical arrangement, the packing is more compact with a well-defined linker. The flexibility of the Xyl linker clearly prevents CBM36-1 being sandwiched in place within the crystal.

### CE4 domain of Xyl

**Fig 3:**
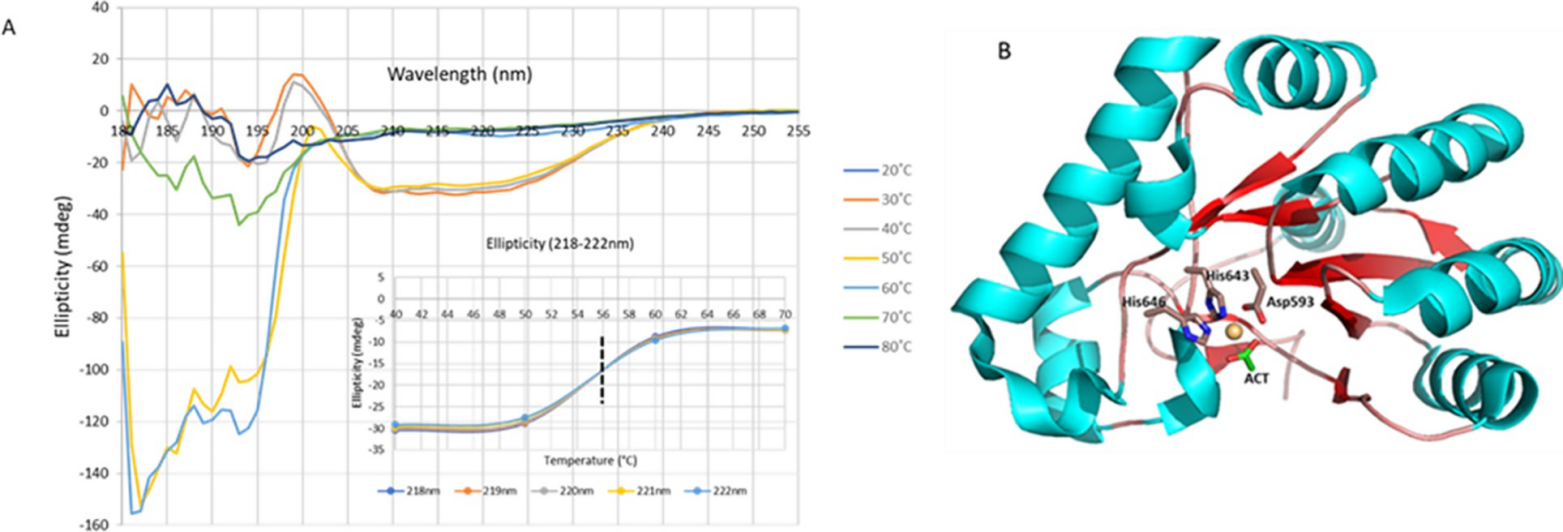
Characterization of Xyl-CE4. **A)** Far-UV circular dichroism spectrum of Xyl-CE4 domain. The CD signature and thermal scan for Xyl-CE4 at temperatures between 20 and 80°C. Ellipticity at increasing temperature (insert) identifies a melting point of 56°C. **B)** The crystal structure of Xyl-CE4 showing α-helices (cyan), β-strands (red), Co^*2+*^ (brown) and acetate (green). The residues coordinating the metal ion are shown as sticks.

The CD spectrum of purified Xyl-CE4 showed a mainly α-helical protein. CD spectra recorded between 20 and 80°C yielded a melting temperature of 56°C (Fig 3A). The esterase activity of Xyl-CE4 was investigated spectrophotometrically by monitoring the release of *para*-nitrophenol (*p-*NP) from 200 μL of 0.5 mM *p*-NPA at 405 nm and 30°C. Xyl-CE4 was active between pH 6.5 and 8.5 with maximum activity at pH 7.5. The enzyme retained 95 and 64% activity after 72 h incubation at pH 7.5 and 7.0 respectively (S1 Fig 3A in S1 File). Catalytic activity of Xyl-CE4 between 20 and 80°C indicated a temperature optimum of 45°C (S1 Fig 3B in S1 File). The effect of divalent metal ions on Xyl-CE4 activity was assayed at 30°C by removing metal ions with 1 mM EDTA and adding divalent ions before assessing enzyme activity. EDTA treatment reduced enzyme activity to 0% of the untreated sample (0.2 μg Xyl-CE4 in 50 mM Tris pH 8.0). Addition of 10 mM Co^2+^, Mg^2+^ and Cu^2+^ restored the enzyme activity to 133, 122 and 110% of the original activity, respectively. By contrast, 10 mM Mn^2+^, Ni^2+^, Zn^2+^ and Ca^2+^ respectively only restored activity to 87, 76, 64 and 34% (S1 Fig 4 in S1 File). A single metal binding site identified in the crystal structure of Xyl-CE4 was correspondingly interpreted as Co^2+^ without further corroboration (Fig 3B). Zn^2+^, the optimal ion for most CE4 enzymes [22], decreased the activity of Xyl-CE4 emphasizing the importance of identifying the optimal metal ions for each metalloenzyme.

Xyl-CE4 at a concentration of 10 mg/mL in 20 mM Tris pH 7.5, 10 mM NaCl was crystallised in 8% (w/v) PEG 4000. X-ray diffraction data to 2 Å resolution collected on a Rigaku diffractometer was processed using HKL3000 [23]. Xyl-CE4 crystal structure (PDB ID: **7AX7**) was solved by molecular replacement using the coordinates of the crystal structure of a family 4 acetyl xylan esterase from *Clostridium thermocellum* (PDB ID: **2C71**) [24] as a search model. Data collection and refinement statistics for all crystal structures are summarised in Table 1.

The Xyl-CE4 domain encompasses 201 amino acid residues folded into eight α-helices, four β-strands and several extended loops that together create a distorted (α/β)-fold in which the β-strands form a curved inner β-sheet surrounded by the α-helices (Fig 3B). The electron density of Xyl-CE4 reveals a metal ion bound through a distorted octahedral coordination by His643, His647, Asp593, a water molecule and an acetate ion. Metal-ion dependent hydrolases typically use metal ions to polarise catalytic residues for nucleophilic attacks on their substrates [24].

### Interdomain interactions in Xyl

Possible molecular recognition between Xyl domains was investigated by isothermal titration calorimetry (ITC) for domain combinations GH11/CBM36-1, GH11/CE4, and CE4/CBM36-1. Ten-fold higher protein concentrations were used for the injected ligand (in syringe) compared to the target (in vessel). Target protein concentrations were in the range of 10 μM to detect potentially weak interactions. However, heats of interaction were not observed for any of the interactions tested (data for GH11/CBM36-1 is shown in S1 Fig 5 in S1 File). We interpreted this lack of interaction to indicate that the individual Xyl domains possibly do not directly interact with each other.

Summarily, individual and combined domains of the multidomain enzyme Xyl were characterised. No evidence of molecular interactions between domains was obtained confirming the prior notion of CBM36 domains not interacting with other protein domains. The increase or decrease in GH11 catalysis when either one or both CBM36 domains are deleted would thus depend on the effective GH11 domain concentration on the polysaccharide surface; the ease with which the GH11 domain targets the polysaccharide substrate; and potentially how the CBM36 domain(s) assist in the disruption of the polysaccharide structure, rather than the interaction of CBM36 domains with catalytic domain. Therefore, CBM36 domains do not enhance the interaction of catalytic domains with their substrate beyond a linker-based co-localization of substrate and catalytic domain. Xyl exemplifies a “beads-on-a-string” protein where the individual domains are “beads” each connected by a linker (string), with each domain retaining its individual function- making the synthesis of novel super enzymes for rapid and efficient degradation of bio-waste to biofuels within reach.

## Materials and methods

### Chemicals, reagents, strains, plasmids, and gene

The restriction enzymes, BamHI, NotI, NcoI and XhoI were purchased from New England Biolabs; GeneJET Plasmid Mini-Prep and PCR Purification kits, GelRed nucleic acid stain and GeneRuler 1 kb DNA Ladder from Thermo-Scientific; Precision plus protein marker from Bio-Rad; and the *E*. *coli* strains DH5α and BL21 CodonPlusRIL from Stratagene. The *xyl* gene coding a 787 amino acid multidomain xylanase Xyl (NCBI GenBank accession number **AMO13186**) from the metagenomic library of the hindgut symbionts of *T*. *trinervoides* [12] was obtained from the South African Centre for Scientific and Industrial Research (CSIR), Pretoria, South Africa.

### Primer design and polymerase chain reaction (PCR)

Protein domains were annotated using the NCBI conserved domains database [25] and the signal peptide delimited by SignalP 4.1 [26]. Reverse and forward primers gene fragments bearing restriction enzyme cut sites were designed to flanked individual and multiple domains (S1 Table 1 in S1 File) and used for PCR amplification of specific DNA fragments. The amplified gene fragments from the *Xyl gene* corresponded to CBM36-1, CBM36-2 and Xyl-CE4, were used to generate plasmid constructs. The same primers used to generate plasmid gene fragments were also used to screen the recombinant plasmids for insertion of genes of interest by PCR. Standard PCR reactions were performed in final volumes of 50 μl containing template DNA (10–100 ng), 1 x reaction buffer, 200 μM of each dNTP, 1 μM of each primer and 1 U Phusion® High-Fidelity DNA Polymerase in a T100™ Thermal Cycler (Bio-Rad laboratories, Hercules, CA, USA) according to Phusion® High-Fidelity DNA Polymerase manufacturer protocol.

### Restriction digest of plasmid DNA and PCR products

Restriction enzymes corresponding to the restriction enzyme site on specific forward and reverse primers used to amplify DNA fragments were also used to restrict the DNA fragments and to linearise plasmid DNA for recombination. Same restriction enzymes used for generation of recombinant plasmids were also used to verify plasmid constructs after recombination for insertion of gene of interest. The desired amount of DNA, varying from 200 ng to 1 μg, was treated with the appropriate amount of restriction enzyme (1–5 U RE/μg DNA depending on the purpose of the restriction digest). Restriction digest reactions were incubated at 37°C for 30 min. Double restriction digests wherein two restriction enzymes are used to restrict the same DNA were performed when necessary.

### Agarose gel electrophoresis

PCR and restriction digest products as well as isolated plasmid DNA were analysed and assessed for quality and yield by agarose gel electrophoresis. This was achieved by mixing 5 μL DNA sample with 1 μL GelRed (Thermo Scientific, Waltham, Massachusetts, USA) and loading onto a 1% agarose gel in a Mini-Sub cell GT cell (Bio-Rad laboratories, Hercules, CA, USA) containing TAE buffer. DNA samples were electrophoresed by applying an electric field of 90 V for 45 min. The DNA bands were visualized on a Molecular Imager Gel Doc XR+ UV transilluminator with Image lab software (Bio-Rad laboratories, Hercules, CA, USA). DNA fragments of interest were extracted from gel slices using the GeneJet gel extraction kit per manufacture’s protocol.

### Ligation of DNA fragments and linearized plasmids

Vector backbones and the desired DNA fragments restricted with the same enzymes were ligated using T4-ligase or T4-quick ligase (NEB). For a standard ligation reaction, 50 ng of dephosphorylated vector DNA was incubated with a threefold molar excess of insert at room temperature overnight for T4-ligase or at 25°C for 30 min for T4-quick ligase. After incubation, ligation mixtures were transformed into competent *E*. *coli* DH5α cells for plasmid DNA propagation and spread on LB agar plates containing appropriate antibiotic.

### Transformation of plasmid DNA into competent bacteria cells

Generally, 50 μl of chemically competent *E*. *coli* cells were transformed with 25 to 50 ng of plasmid DNA. The frozen competent cells were thawed on ice, mixed with plasmid DNA, incubated for 30 min on ice, heat shocked at 42°C for 45 s and chilled on ice for 2 min. The cells were supplemented with 800 μL of LB medium and incubated at 37°C for 1 h. One tenth of the incubated cells culture was spread on LB agar plates with appropriate antibiotics and incubated overnight at 37°C. As a control, untransformed competent cells were plated on agar plates with and without antibiotics and incubated alongside the experimental plates.

### Sequencing of plasmid DNA

To confirm the DNA sequence of recombinant plasmid constructs, the constructs were sequenced by Sanger sequencing at the Inqaba sequencing facility (Inqaba Biotec, South Africa) using the appropriate sequencing primer. Raw sequencing data was analysed using the Heracle BioSoft, DNA Sequence Assembler v4 (2013).

### Protein production and purification

Recombinant plasmids bearing genes of interest were used to transform competent BL21 CodonPlusRIL *E*. *coli* and cultured at 37°C in LB with 100 μg/mL ampicillin for pGEX-6P-1, 100 μg/mL ampicillin and 34 μg/mL chloramphenicol for pET20b or 50 μg/mL kanamycin and 34 μg/mL chloramphenicol for pET28a. At OD_600_ = 0.6, protein production was induced with 0.5 mM IPTG at 28°C for 16 h. Cells were collected by centrifugation at 6 000 x *g*, 4°C, 15 min and disrupted by sonication in 25 mM Tris-HCl pH 8.0, 300 mM NaCl. After centrifugation at 37 000 x *g* at 4°C for 45 min, the supernatant was transferred to either Co^2+^-bearing immobilized metal affinity chromatography (IMAC) resin (G-Biosciences, St. Louis, MI, USA) to bind His_6_-tagged target proteins, or GS resin (Novagen) for GST-tagged proteins. Non-specifically bound proteins were eluted with lysis buffer supplemented either with 10 mM imidazole or 2 mM reduced glutathione for 6xHis-tagged proteins and GST-tagged proteins respectively. All remaining 6xHis-tagged proteins on resins were eluted with 250 mM imidazole whereas GST-tagged proteins with agitated overnight with 3C protease. Cleaved off GST remained on resins and protein of interest was collected as flow through. All fractions were analysed by SDS-PAGE, concentrated, and further purified by size exclusion chromatography using a Superdex 200 10/300 GL column on an ÄKTA 900 chromatography system (GE Healthcare, Chicago, IL, USA) exchanging the buffer by 10 mM Tris-HCl pH 8.0, 10 mM NaCl. Sample purity was assessed by SDS-PAGE and samples were stored at 4°C.

### Circular dichroism (CD) spectroscopy

Fingerprint CD spectra for Xyl-CBM36 and Xyl-CE4 domains were recorded at 20°C on a Chirascan (Applied Photophysics, Leatherhead, UK). Domain stabilities were assessed by recording CD spectra at intervals 10°C between 20 and 80°C using 4 μM protein in 25 mM Tris-HCl pH 8.0, 25 mM NaCl after 5 min incubation. The thermal unfolding profile of Xyl-CE4 at 220 nm was extracted and fitted to a Boltzmann sigmoidal curve.

### Xyl-CE4 activity assay

Acetyl esterase activity for Xyl-CE4 was determined spectrophotometrically by quantifying the release of *para*-nitrophenol (*p*-NP) from *p*-NPA at 405 nm and 30°C. Assay mixes contained 0.5 mM *p*-NPA (Merck, Darmstadt, Germany), 50 mM Tris-HCl pH 8, and 0.2 μg Xyl-CE4. Temperature dependence of acetyl esterase activity was determined between 20 and 80°C, the pH optimum at 30°C between pH 3 to 10 using 50 mM phosphate citrate pH 3 to 5, 50 mM MES pH 5.5 to 6.5, 50 mM Tris-HCl pH 7 to 9, and 50 mM CHES pH 9.5 to 10. The pH stability between pH 3 and 10 was determined by measuring residual activity after incubating Xyl-CE4 for 72 h at 30°C. The effect of divalent cations (10 mM of Ca^2+^, Fe^2+^, Co^2+^, Mn^2+^, Cs^2+^, Mg^2+^, Ni^2+^ and Cu^2+^) on Xyl-CE4 activity was assayed at 30°C. Metal ions were removed with 1 mM EDTA and the protein washed with 50 mM Tris-HCl pH 8.0. Divalent ions were added to the EDTA treated protein, which was assayed and compared to the untreated enzyme. All assays were done in triplicates.

### Crystallization and structure solution

Protein samples were crystallized by sitting-drop vapour-diffusion using commercial crystallization screens combining 1 μL protein and 1 μL reservoir solution against 80 μL reservoir solution at 18°C. Crystal hits were iteratively optimized. Single crystals were cryoprotected with 20 to 25% (v/v) PEG 400 in reservoir fluid. X-ray diffraction data were collected at 100 K either on a rotating copper anode diffractometer (MicroMax-007HF plus Saturn 944HG CCD, Rigaku, Japan) at the University of Cape Town, South Africa or on beamline ID29 of the European Synchrotron Radiation Facility (ESRF), Grenoble, France and processed with the HKL3000 software suite [23] or the Grenoble automatic data processing system (GrenADeS) [27].

Crystal structures were solved by molecular replacement (MR) in Phaser [28]. Structures were rebuilt using Phenix AutoBuild [29] and manually corrected in COOT [30] and refined in Phenix. PyMOL [31] was used to analyse and present structure images.

Protein-protein interactions were analyzed in PBS at 25°C using an ITC200 microcalorimeter (Microcal/GE Healthcare, USA). The titrant (protein in syringe) concentration was invariably ten-fold that of the titrand (protein in sample cell) (S1 Table 2 in S1 File). As control, the titrant was titrated into the titrand buffer. For each run, eighteen 2 μl aliquots of titrant were injected into 150 μl of titrand. Experiments were repeated twice. Integrated heat data were analysed with a single binding-site model in Origin (Microcal Software, Northhampton, MA, USA).

### Accession number

PDB ID: **7ZSZ, 7AYP, 7AY3, 7AX7**

## Supporting information

S1 File Primers, purification and characterization of Xyl domains. S1 Fig 1: Production and Co^2+^-NTA purification of Xyl. Xyl was eluted with increasing concentration of imidazole and elution fractions pulled and concentrated. Concentrated protein showed two major bands and degraded completely with overnight storage even in the presence of protease inhibitor cocktails. S1 Fig 2: GST-fusion and size exclusion chromatography purification of CBM36-2. A) GST- CBM36-1 and GST-CBM36-2 treated with 3C protease. Lanes 1 and 3: Protein samples after overnight treated with 3C protease at 4°C. Lanes 2 and 4 show eluted protein from overnight cleavage. Lanes R, represents GS resins after elution. The large blob of protein is the GST tag left on the resins. Protein samples from lanes 2 and 4 were separately concentrated and further purified by size exclusion chromatography. B). CBM36-2 purified from size exclusion chromatography. Lane 1 is concentrated protein before loading on GS column. Lane 2 shows separated impurity. The read boxes represent protein on chromatogram peaks corresponding to the size of CBM36-2. The green box represents CBM36-2 concentrated for crystallization. S1 Fig 3: Activity of Xyl-CE4 with increasing pH and temperature identifies optimum pH and temperature at 7.5 and 45°C respectively. A) pH activity measured from the release of *para*-nitrophenol (*p-*NP) from 200 μL of 0.5 mM *p*-NPA at 405 nm and 30°C. Blue curve optimum activity assay and orange curve is activity of enzyme after 72 h of incubation. B) Temperature profile of Xyl-CE4 at increasing temperature measured from the release of *p-*NP from 200 μL of 0.5 mM *p*-NPA at 405 nm and pH 7.5. S1 Fig 4: Effect of divalent metal ions on Xyl-CE4 catalysis assayed at 30°C by removing metal ions with 1 mM EDTA and adding divalent ions before assessing enzyme activity. EDTA treatment reduced enzyme activity to 0% of the untreated sample (XylCE4_purified: 0.2 μg Xyl-CE4 in 50 mM Tris pH 8.0). Co^2+^ is most highly activating. S1 Fig 5: Analysis of GH11/CBM36-1 interaction by ITC using an ITC200 microcalorimeter (Microcal/GE Healthcare, USA). 10 μM CBM36-1 in the syringe, was titrated into 1 μM GH11 at 25°C in PBS buffer. No heats of interactions are observed. S1 Table 1. Primers used for cloning and sequencing recombinant gene fragments. S1 Table 2. Concentration of proteins used for ITC experiments.
(DOCX)
