# Supplementary material for: Structural and biophysical characterization of the multidomain xylanase Xyl
Source: PLoS One. 2022 Jun 3;17(6):e0269188. doi: 10.1371/journal.pone.0269188 (PMC9165906; doi:10.1371/journal.pone.0269188)
Supplement: S1 File — S1 Fig 1: Production and Co2+-NTA purification of Xyl. Xyl was eluted with increasing concentration of imidazole and elution fractions pulled and concentrated. Concentrated protein showed two major bands and degraded completely with overnight storage even in the presence of protease inhibitor cocktails. S1 Fig 2: GST-fusion and size exclusion chromatography purification of CBM36-2. A) GST- CBM36-1 and GST-CBM36-2 treated with 3C protease. Lanes 1 and 3: Protein samples after overnight treated with 3C protease at 4°C. Lanes 2 and 4 show eluted protein from overnight cleavage. Lanes R, represents GS resins after elution. The large blob of protein is the GST tag left on the resins. Protein samples from lanes 2 and 4 were separately concentrated and further purified by size exclusion chromatography. B). CBM36-2 purified from size exclusion chromatography. Lane 1 is concentrated protein before loading on GS column. Lane 2 shows separated impurity. The read boxes represent protein on chromatogram peaks corresponding to the size of CBM36-2. The green box represents CBM36-2 concentrated for crystallization. S1 Fig 3: Activity of Xyl-CE4 with increasing pH and temperature identifies optimum pH and temperature at 7.5 and 45°C respectively. A) pH activity measured from the release of para-nitrophenol (p-NP) from 200 μL of 0.5 mM p-NPA at 405 nm and 30°C. Blue curve optimum activity assay and orange curve is activity of enzyme after 72 h of incubation. B) Temperature profile of Xyl-CE4 at increasing temperature measured from the release of p-NP from 200 μL of 0.5 mM p-NPA at 405 nm and pH 7.5. S1 Fig 4: Effect of divalent metal ions on Xyl-CE4 catalysis assayed at 30°C by removing metal ions with 1 mM EDTA and adding divalent ions before assessing enzyme activity. EDTA treatment reduced enzyme activity to 0% of the untreated sample (XylCE4_purified: 0.2 μg Xyl-CE4 in 50 mM Tris pH 8.0). Co2+ is most highly activating. S1 Fig 5: Analysis of GH11/CBM36-1 interaction by ITC using [file pone.0269188.s001.docx]

**Supplementary Information**

**S1 Table 1:** Primers used for cloning and sequencing recombinant gene fragments

| **Name of primer** | **Primer sequence (5’…3’)** |
| --- | --- |
| pGEX-6P-1_*cbm36-1* Fd (BamHI) | ACTTGGGGATCC**GGCAACACCGGTAGTCAAACG** |
| pGEX-6P-1_*cbm36-1* Rv (NotI) | AATTAGCGGCCGCTCA**CGATGATTCATTGCCACCAAC** |
| pGEX-6P-1_*cbm36-2* Fd (BamHI) | ACTTGGGGATCC**GGTGGCCAGGCTCAAAGC** |
| pGEX-6P-1_*cbm36-2* Rv (NotI) | AATTAGCGGCCGCTCA**CGTTTGAACCGGCATTGTTGC** |
| pET28a_*ce4* Fd (NcoI) | ACTACCCATGG**GCAATGAAAAACTGATCG** |
| pET28a_*ce4* Rv (XhoI) | TATACCTCGAG**TTGACCGTTAGCGTTTGAC** |
| T7 sequencing primer Fd | TAATACGACTCACTATAGGG |
| T7 sequencing primer Rv | GCTAGTTATTGCTCAGCGG |

**S1 Table 2:** Concentration of proteins used for ITC experiments

| **Titrant (Protein in syringe)** | **Titrand (protein in vessel)** |
| --- | --- |
| CBM36-1 (10 mg/ml) = 714 µM | GH11 (1 mg/ml) = 38.5µM |
| CE4 (10 mg/ml) = 437.8 µM | CBM36-1 (0.6 mg/ml) = 42.8 µM |
| CE4 (10 mg/ml) = 437.8 µM | GH11 (1.1 mg/ml) = 42 µM |


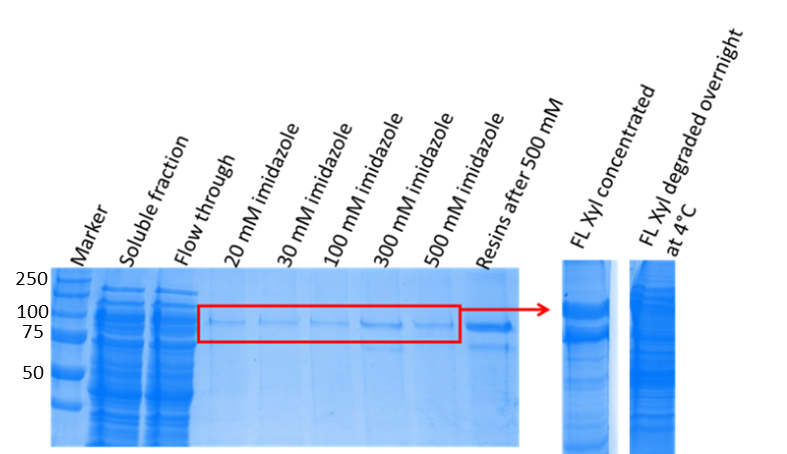


**S1 Fig 1:** Production and Co^2+^-NTA purification of Xyl. Xyl was eluted with increasing concentration of imidazole and elution fractions pulled and concentrated. Concentrated protein showed two major bands and degraded completely with overnight storage even in the presence of protease inhibitor cocktails.


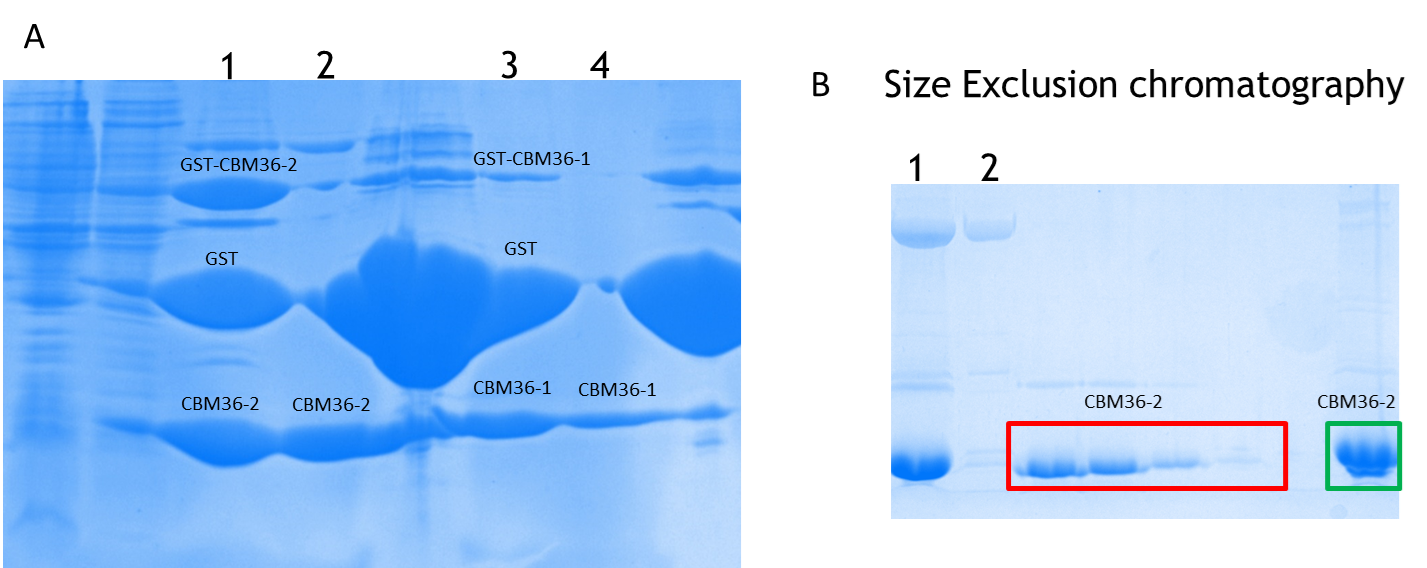


**S1 Fig 2:** GST-fusion and size exclusion chromatography purification of CBM36-2. A) GST‑ CBM36-1 and GST-CBM36-2 treated with 3C protease. Lanes 1 and 3: Protein samples after overnight treated with 3C protease at 4 °C. Lanes 2 and 4 show eluted protein from overnight cleavage. Lanes R, represents GS resins after elution. The large blob of protein is the GST tag left on the resins. Protein samples from lanes 2 and 4 were separately concentrated and further purified by size exclusion chromatography. B). CBM36-2 purified from size exclusion chromatography. Lane 1 is concentrated protein before loading on GS column. Lane 2 shows separated impurity. The read boxes represent protein on chromatogram peaks corresponding to the size of CBM36-2. The green box represents CBM36-2 concentrated for crystallization.


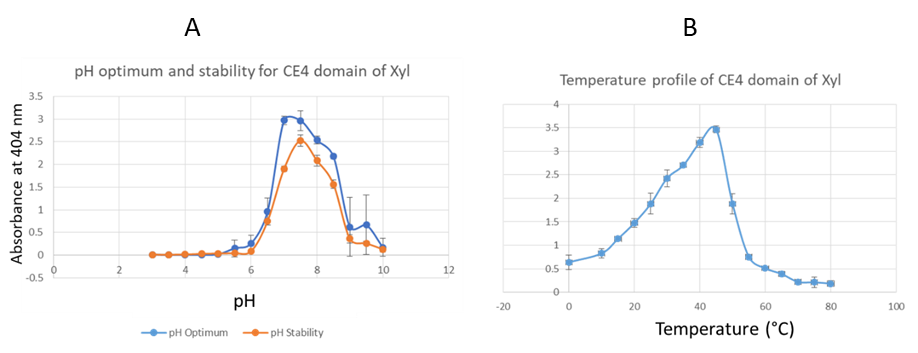


**S1 Fig 3**: Activity of Xyl-CE4 with increasing pH and temperature identifies optimum pH and temperature at 7.5 and 45 °C respectively. A) pH activity measured from the release of *para*-nitrophenol (*p-*NP) from 200 μL of 0.5 mM *p*-NPA at 405 nm and 30 °C. Blue curve optimum activity assay and orange curve is activity of enzyme after 72 h of incubation. B) Temperature profile of Xyl-CE4 at increasing temperature measured from the release of *p-*NP from 200 μL of 0.5 mM *p*-NPA at 405 nm and pH 7.5. Both pH and temperature assays were performed in triplicates and standard deviation error bars plotted.


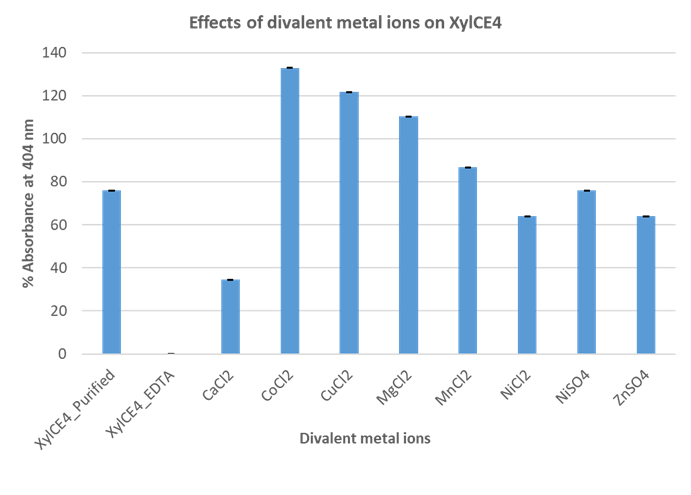


**S1 Fig 4**: Effect of divalent metal ions on Xyl-CE4 catalysis assayed at 30 °C by removing metal ions with 1 mM EDTA and adding divalent ions before assessing enzyme activity. EDTA treatment reduced enzyme activity to 0 % of the untreated sample (XylCE4_purified: 0.2 μg Xyl-CE4 in 50 mM Tris pH 8.0). Co^2+^ is most highly activating. All assays were performed in triplicate and standard deviation error bars indicated.

**
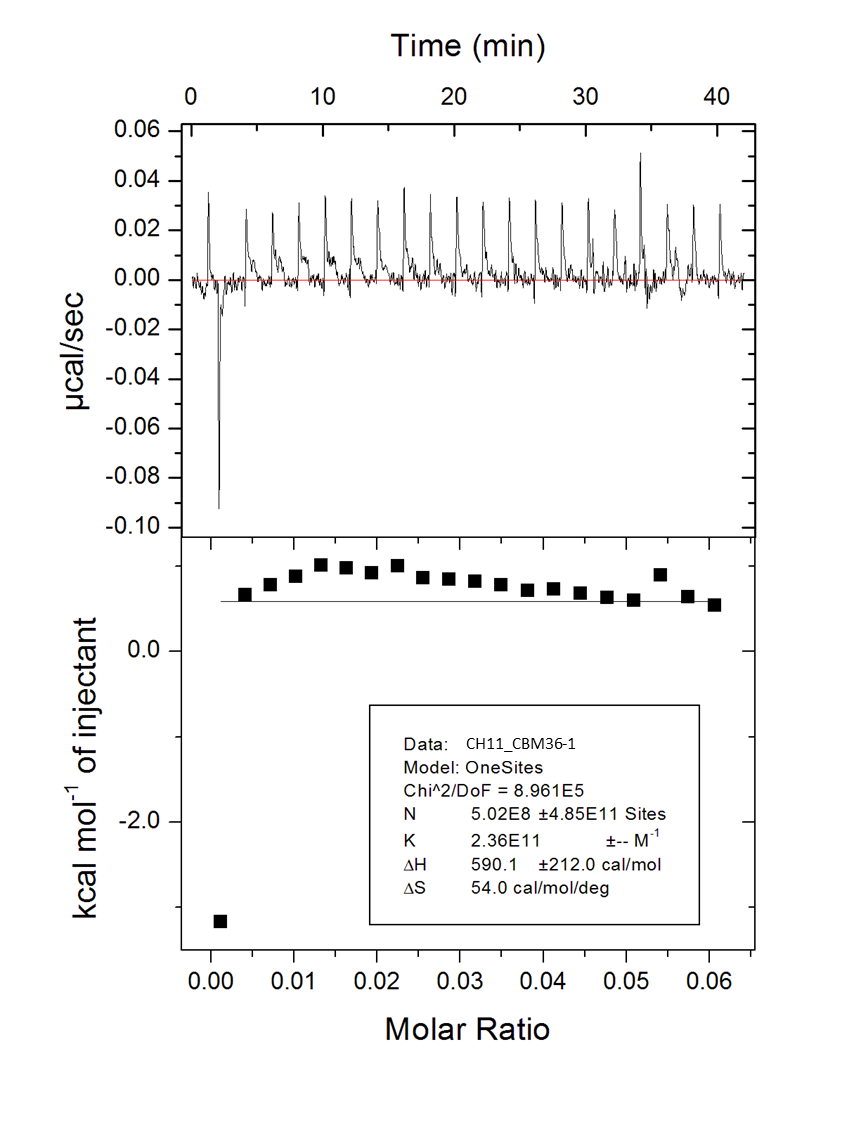
**

**S1 Fig 5:** Analysis of GH11/CBM36-1 interaction by ITC using an ITC200 microcalorimeter (Microcal/GE Healthcare, USA). 10 µM CBM36-1 in the syringe, was titrated into 1 µM GH11 at 25 °C in PBS buffer. No heats of interactions are observed.
